# Gender-dependent difference in serum paraoxonase 1 levels of Hanwoo, Korean native cattle, and a positive association with meat quality

**DOI:** 10.5713/ajas.18.0138

**Published:** 2018-08-27

**Authors:** Jihyun Park, Jiwoo Kim, Sungwon Hwang, Ki Young Chung, Inho Choi, Chang Bon Choi, Jihoe Kim

**Affiliations:** 1 Department of Medical Biotechnology, Yeungnam University, Gyeongsan 38541, Korea; 2 Hanwoo Experiment Station, National Institute of Animal Science, RDA, Pyongchang 25340, Korea

**Keywords:** Hanwoo, Serum paraoxonase 1, Gender-dependent Difference, Association with Meat Quality

## Abstract

**Objective:**

Paraoxonase 1 (PON1), a calcium-dependent serum enzyme, has been shown to be involved in lipid metabolism. In this study, we examined the putative correlation of the serum PON1 level of Hanwoo, Korean native cattle, with gender and meat quality grade.

**Methods:**

PON1 levels were estimated by determining the arylesterase and paraoxonase activities (AE and PO, respectively) in serum samples from Hanwoo individuals (n = 56). Serum PON1 levels were analyzed in different gender groups (female [n = 21], castrated male [n = 17], and male [n = 18]), and meat quality grades (≥1 [n = 23], 2 [n = 21], and 3 [n = 12]).

**Results:**

Serum PON1 levels were similar in female (AE = 120±55 U/mL, PO = 84±43 mU/mL) and castrated male (123±44 U/mL, PO = 89±30 mU/mL), while male showed a significantly lower level (AE = 65±43 U/mL, PO = 44±34 mU/mL). Furthermore, analysis of serum PON1 levels in three different grades of meat quality showed similar levels in the grades ≥1 (AE = 118±49 U/mL, PO = 84±37 mU/mL) and 2 (AE = 116±54 U/mL, PO = 82±43 mU/mL), while the level was significantly lower in the grade 3 (AE = 58±35 U/mL, PO = 39±27 mU/mL) of lower meat quality.

**Conclusion:**

We discovered the gender-dependent differences in serum PON1 levels of Hanwoo and a positive association of the serum PON1 level with meat quality. Results in this study suggest that PON1 would be a useful serum marker for preliminary screening of Hanwoo individuals with high-quality meat and applicable for genetic improvement.

## INTRODUCTION

Paraoxonase 1 (PON1), a calcium-dependent serum enzyme, is primarily synthesized in the liver and secreted into blood, where it is associated with high density lipoprotein [1]. Although the physiological role of PON1 has not been clearly elucidated, PON1 was shown to be involved in a drug metabolism by hydrolyzing toxic metabolites of organophosphorus compounds used as insecticides and nerve agents [2–4]. PON1 also showed antioxidant activity preventing oxidation of lipids in low-density lipoproteins and high-density lipoproteins [5,6]. Moreover, serum PON1 levels were estimated to be low in obesity, insulin resistance and dyslipidemia [7], indicating the involvement of the enzyme in lipid metabolism. Polymorphic variants of human PON1 were reported to be significantly different in the enzyme activity of PON1 showing a positive association with the fatty acid composition of the adipose tissue [8]. Another study demonstrated an inverse correlation of PON1 activity with the thickness of epicardial adipose tissue, which suggested a possible role of PON1 in the regulation of the lipid composition in body [9].

Hanwoo is a type of Korean native cattle and primary source of beef in Korea. Hanwoo beef is preferred over imported beef in Korean markets even though its price is twice as expensive, because consumers believe that Hanwoo beef have better quality [10,11]. It was reported that Hanwoo beef have greater scores of tenderness, juiciness, and flavor than imported beef from the United States and Australia [12]. Korea Institute for Animal Products Quality Evaluation (KAPE) established a beef carcass grading system consisting three levels of yield grade evaluating meat amount (A, B, and C) and five levels of meat quality grade (higher to lower quality: 1^++^, 1^+^, 1, 2, and 3) [13]. Several factors, including marbling score, meat color, fat color, and firmness and texture are considered to determine the meat quality grade. However, marbling score is the most dominant determinant for beef meat grading, since fat and fatty acids importantly contribute to eating quality. Many studies have reported that palatability improves as marbling increases [14–16]. In addition, marbling score is the most decisive criterion affecting purchase decisions, not only in Korea, but also in other countries [17,18].

Although most studies about PON1 focused on the human enzyme, PON1 was also found in bovine serum and its activity was shown to be a useful marker of diseases in dairy cows [19,20]. However, PON1 has been rarely investigated in cattles for meat production. Considering the possible role of PON1 in the regulation of the lipid composition and the most dominant determinant, marbling score, for Hanwoo meat quality grading, we examined the putative correlation of the serum PON1 level with gender and meat quality grade.

## MATERIALS AND METHODS

### Materials and serum samples

All chemicals were purchased from Sigma Aldrich, unless otherwise mentioned. Blood samples from 56 Hanwoo individuals (2 to 3-year old female, n = 21; 2-year old males, n = 18; 2-year old castrated male, n = 17) were collected in sterile bottles from a local slaughter-house in Pyeongchang, Kangwon Do, Republic of Korea. Meat quality grades were evaluated according to the KAPE carcass grading system [13]. Serum was isolated from collected blood samples and sterilized, as previously described [21], and stored at −20°C until use. Protein concentrations of serum samples were determined by Bradford assay [22].

### Paraoxonase 1 activity assays

The arylesterase and paraoxonase activities of PON1 were measured spectrophotometrically using the substrates phenylacetate and paraoxon (*O*, *O*-diethyl *O*-(4-nitrophenyl) phosphate), respectively [23]. Briefly, the arylesterase assay contained 1 mM phenylacetate in 20 mM Tris-HCl pH 8.0, 1 M NaCl, and 1 mM CaCl_2_ at room temperature. The reaction was started by the addition of serum samples, and the formation of the reaction product, phenol, was followed at 270 nm (ɛ270 nm = 1.31 mM^−1^ cm^−1^). The paraoxonase assay contained 1 mM paraoxon in the same buffer as described above. The addition of serum samples started the reaction and the formation of the reaction product, *p*-nitrophenol, was followed at 412 nm (ɛ412 nm = 17.1 mM^−1^ cm^−1^). Enzyme activities were calculated from the initial slope of product formation and expressed in U (μmol/min·mL) and mU (nmol/min·mL) for arylesterase and paraoxonase activities, respectively.

### Statistical analysis

Serum samples were grouped into three different genders of female (n = 21), castrated male (n = 17) and male (n = 18), and three different beef meat quality grades of ≥1 (n = 23), 2 (n = 21), and 3 (n = 12). The average PON1 activities were expressed as the means±standard error. Differences in serum PON1 activities among genders and beef meat quality grades were analyzed by unpaired Student’s t-test. A p value <0.01 was considered statistically significant.

## RESULTS AND DISCUSSION

**Figure 1:**
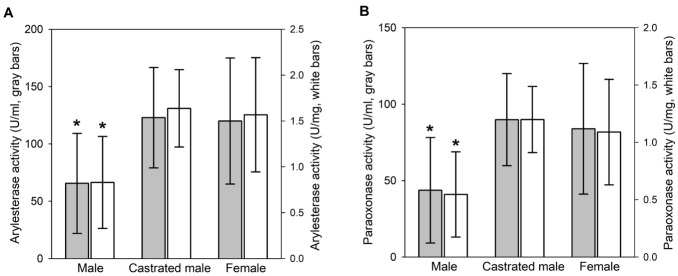
Gender-dependent differences in serum paraoxonase 1 (PON1) levels. PON1 enzyme acitivities in Table 1 were grouped into different groups of genders. Mean values of gender groups are compared for arylesterase (A) and paraoxonase (B) acitivities. Gray bars are for volume activities (U/mL or mU/mL) and white bars are for activities normalized by serum proteins concentrations (U/mg or mU/mg). * The serum PON1 level in male is significantly lower (statistical significance of p<0.001) than the levels in female and castrated male.

Serum samples were collected from 56 Hanwoo individuals, and grouped into three different gender groups (female, n = 21; castrated male, n = 17; male, n = 18). The serum PON1 levels were then estimated by determination of the arylesterase and paraoxonase activities of PON1 (AE and PO, respectively) in serum samples (Table 1), as described in the materials and methods. The AE was determined to be highly variable in serum samples within the range of ~20 to 240 U/mL, although serum protein concentrations were in the range of ~60 to 100 mg/mL. Statistical analyses showed similar AE of 120±55 U/mL and 123±44 U/mL in female and castrated male, respectively (Figure 1A). However, the AE was 65±43 U/mL in male and significantly lower than the activities in the other gender groups. The PO was also determined to be highly variable in serum samples within the range of ~10 to 200 mU/mL. And, the PO was significantly lower in male (44±34 mU/mL) than in female and castrated male (84±43 mU/mL and 89±30 mU/mL, respectively) (Figure 1B). Analyses of AE and PO normalized by serum protein concentration consistently showed lower the PON1 activities in male than in other gender groups (Figure 1A, 1B). These results indicated the gender-dependent difference in serum PON1 levels of Hanwoo.

**Figure 2:**
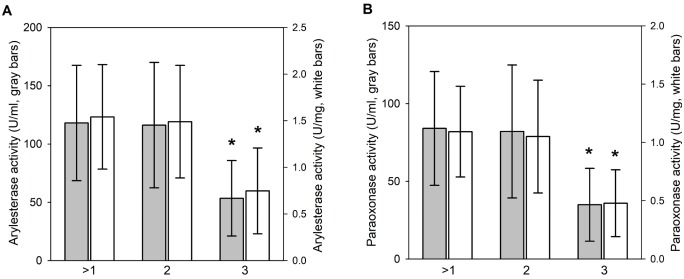
Positive association of serum paraoxonase 1 (PON1) level with meat quality. PON1 enzyme acitivities in Table 1 were grouped into different groups of meat quality grades. Mean values of meat quality groups were compared for arylesterase (A) and paraoxonase (B) acitivities. Gray bars are for volume activities (U/mL or mU/mL) and white bars are for activities normalized by serum proteins concentrations (U/mg or mU/mg). * The serum PON1 level in the grade 3 is significantly lower (statistical significance of p<0.001) than the levels in the grade ≥1 and 2.

The gender-dependent difference in the serum PON1 level of Hanwoo suggested a putative correlation with meat quality, which prompted us to analyze the data in different meat quality groups. Analysis of the PON1 levels in three different meat quality grades (≥1, n = 23; 2, n = 21; 3, n = 12) showed that AE were similar in the grade ≥1 and 2 (118±49 U/mL and 116±54 U/mL, respectively (Figure 2A). While AE was 58±35 U/mL in the grade 3 and significantly lower than the activities in the other meat grade groups (Figure 2A). Consistently, the PO in the grade 3 was 39±27 mU/mL, which was significantly lower than in the grade ≥1 and 2 (84±37 mU/mL and 82±43 mU/mL, respectively) (Figure 2B). AE and PO normalized by serum protein concentration were consistently lower in the grade 3 than in the other meat grade groups (Figure 2A and 2B). The grade ≥1 included mostly females (n = 11) and castrated males (n = 10), and only two males. Interestingly, males in the grade ≥1 showed serum PON1 levels (AE activities = 77 U/mL and 201 U/mL, PE activities = 47 U/mL and 152 U/mL) higher than the average level of the male group (Table 1). Moreover, the grade 3 included mostly males (n = 10), and two females. Serum PON1 levels of two females in the grade 3 (AE activities = 16 U/mL and 34 U/mL, PO activities = 9 mU/mL and 29 mU/mL) were much lower than the average level of the female group (Table 1). The grade 2 included eight females, seven castrated males and six males. The average serum PON1 activity level of males in the grade 2 (AE activity = 52±24 U/mL, PO activity = 34±21 mU/mL) was not significantly different from the average level of the male group.

The mean values of serum PON1 levels obtained in this study were with rather high deviations, which might be due to fixed effect. However, when the same experiment was carried out multiple times with another sets of samples, data analysis showed similar extend of deviations, but with consistent gender-dependent differences in serum PON1 levels and the positive association with meat quality. Therefore, we concluded that the high deviations of mean values were mostly due to a few number of samples with exceptional high or low PON1 levels in each gender group and meat quality grade. These exceptional serum samples implicated that certain genetic or environmental factors could be related with serum PON1 levels. Age, body weight and feeding conditions might affect serum PON1 levels, which could not be considered and analyzed in this study due to the limited sample numbers and insufficiently controlled managing environments. Nevertheless, we could not exclude the data of exceptional high or low PON1 levels for analysis, since they were from rare samples, such as males in the meat grade ≥1 and females in the meat grade 3.

Bovine gender is a well-known factor that affects marbling score and meat quality. Marbling score was shown to be significantly higher in female and castrated male than in male, regardless of bovine species, indicating possible roles of sex hormones in the regulation of fat deposition [24,25]. The gender-dependent difference in serum PON1 levels of Hanwoo indicated a close relation with sex hormones, which was not observed in humans [26]. The positive association of serum PON1 levels with meat quality could be due to more males in the grade 3 than the other meat grade groups. However, a few females in the grade 3 with very low PON1 levels and males in the grade 1 with high PON1 levels suggested that differences in serum PON1 levels might be, not only gender-dependent, but also dependent of a certain genetic factor(s). Certain polymorphic variants of human PON1 showed high enzyme activities and a positive association with the fatty acid composition in the body [8,9]. The positive association of serum PON1 levels with meat quality implicates the PON1 polymorphism that needs to be investigated for the genetic improvement of Hanwoo.

In conclusion, we discovered a gender-dependent difference in serum PON1 levels of Hanwoo, which was significantly lower in male than in female and castrated male. Furthermore, the serum PON1 level was positively associated with meat quality, showing lower levels in the lower meat quality group of the grade 3 than in higher meat quality groups of the grade ≥1 and 2. Results in this study suggest that PON1 would be a useful serum marker for preliminary screening of Hanwoo individuals with high-quality meat and applicable for genetic improvement.

## Figures and Tables

**Table 1:** Summary of PON1 enzyme activities for serum samples grouped into different meat quality grades and genders1)

Meat grade	Gender	Serum protein Conc. (mg/mL)	AE2) (U/mL)	AE3) (U/mg)	PO4) (mU/mL)	PO5) (mU/mg)
≥1 (n = 23)	Castrated male (n = 10)	62	105	1.70	79	1.28
		77	99	1.28	83	1.07
		67	87	1.29	63	0.93
		77	165	2.14	123	1.60
		68	129	1.89	83	1.22
		82	130	1.58	79	0.97
		68	84	1.23	69	1.01
		77	66	0.85	61	0.79
		61	62	1.03	54	0.89
		71	93	1.31	72	1.01
	Male6) (n = 2)	107	77	0.72	47	0.44
		94	201	2.13	153	1.62
	Female (n = 11)	69	173	2.52	112	1.63
		99	162	1.63	111	1.11
		100	244	2.45	192	1.93
		66	105	1.60	78	1.19
		69	60	0.87	44	0.63
		64	142	2.21	100	1.56
		83	114	1.38	87	1.05
		61	131	2.15	76	1.25
		83	132	1.60	71	0.86
		62	26	0.42	21	0.33
		81	117	1.44	73	0.90
2 (n = 21)	Castrated male (n = 7)	59	106	1.80	64	1.08
		52	115	2.22	85	1.66
		102	232	2.27	152	1.48
		83	151	1.80	127	1.52
		83	171	2.07	140	1.70
		69	125	1.80	75	1.09
		108	168	1.56	117	1.09
	Male (n = 6)	56	39	0.69	28	0.49
		77	37	0.48	22	0.29
		100	45	0.45	13	0.13
		85	97	1.15	70	0.82
		80	59	0.74	47	0.60
		99	34	0.34	24	0.24
	Female (n = 8)	60	98	1.63	62	1.03
		68	123	1.82	113	1.68
		98	180	1.84	156	1.59
		75	126	1.68	80	1.08
		81	169	2.09	106	1.31
		88	160	1.82	108	1.22
		75	107	1.43	62	0.83
		63	100	1.60	71	1.13
3 (n = 12)	Male (n = 10)	60	51	0.84	31	0.51
		67	27	0.40	24	0.36
		60	76	1.26	52	0.86
		61	109	1.80	57	0.93
		65	40	0.62	23	0.36
		66	52	0.79	32	0.49
		68	31	0.46	9	0.13
		92	19	0.21	12	0.13
		98	78	0.79	54	0.55
		101	108	1.07	87	0.87
	Female7) (n = 2)	69	34	0.50	29	0.42
		69	15	0.22	9	0.13

PON1, paraoxonase 1; AE, arylesterase activity; PO, paraoxonase activity.

1) Mean values for the groups of genders and meat grades are compared in Figure 1 and 2, respectively.

2), 4) Arylesterase and paraoxonase volume activities, respectively.

3), 5) Arylesterase and paraoxonase activities, respectively, normalized by serum protein concentrations.

6) Males in the grade ≥1 showed serum PON1 levels higher than the average level of the male group.

7) Females in the grade 3 showed serum PON1 levels lower than the average level of the female group.
